# Supplementary figures and images for: Considerations for metabarcoding‐based port biological baseline surveys aimed at marine nonindigenous species monitoring and risk assessments
Source: Ecol Evol. 2020 Feb 7;10(5):2452–65. doi: 10.1002/ece3.6071 (PMC7069299; doi:10.1002/ece3.6071)

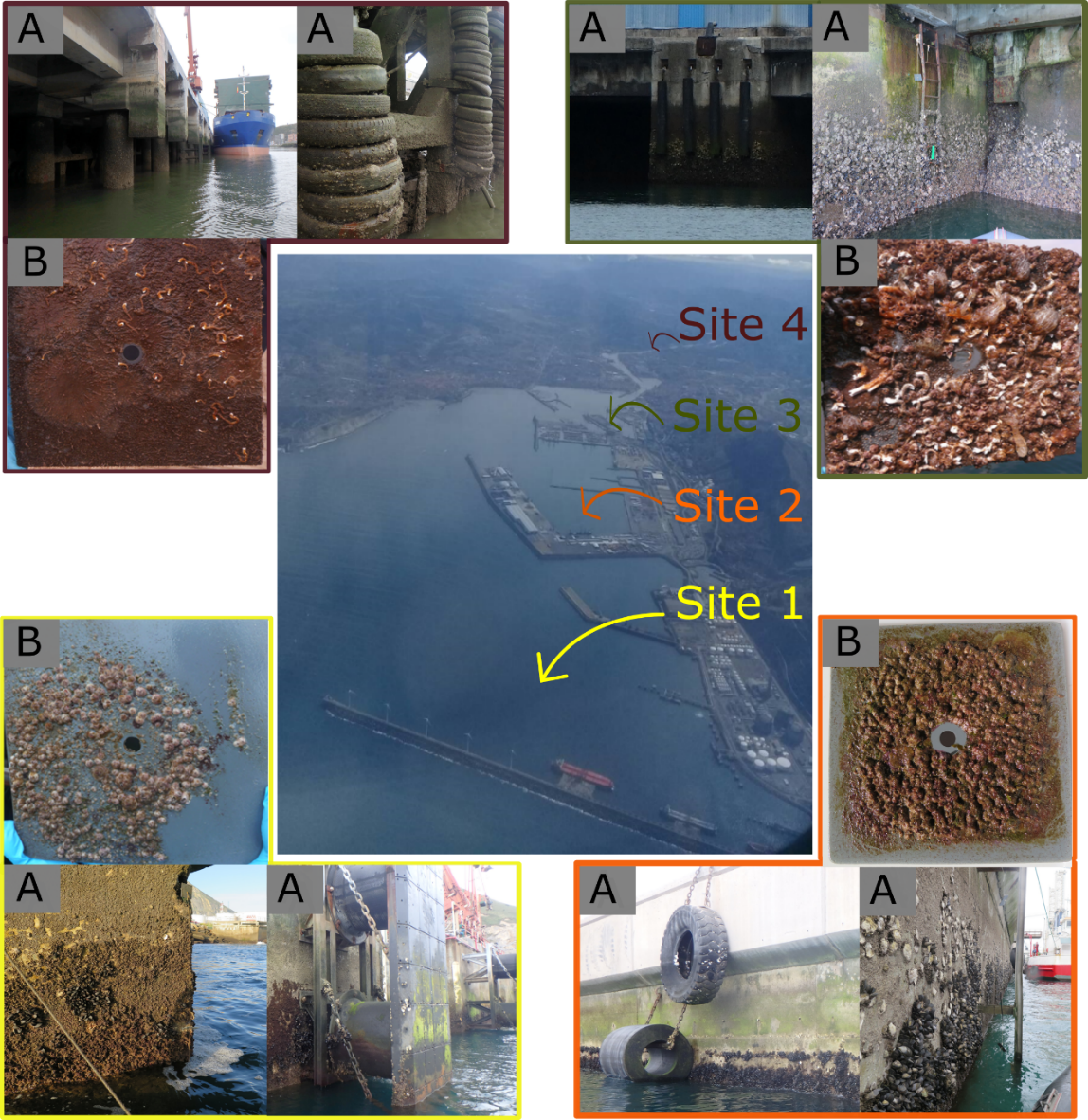

Supplement: Supplementary file 2 [file ECE3-10-2452-s002.tif]

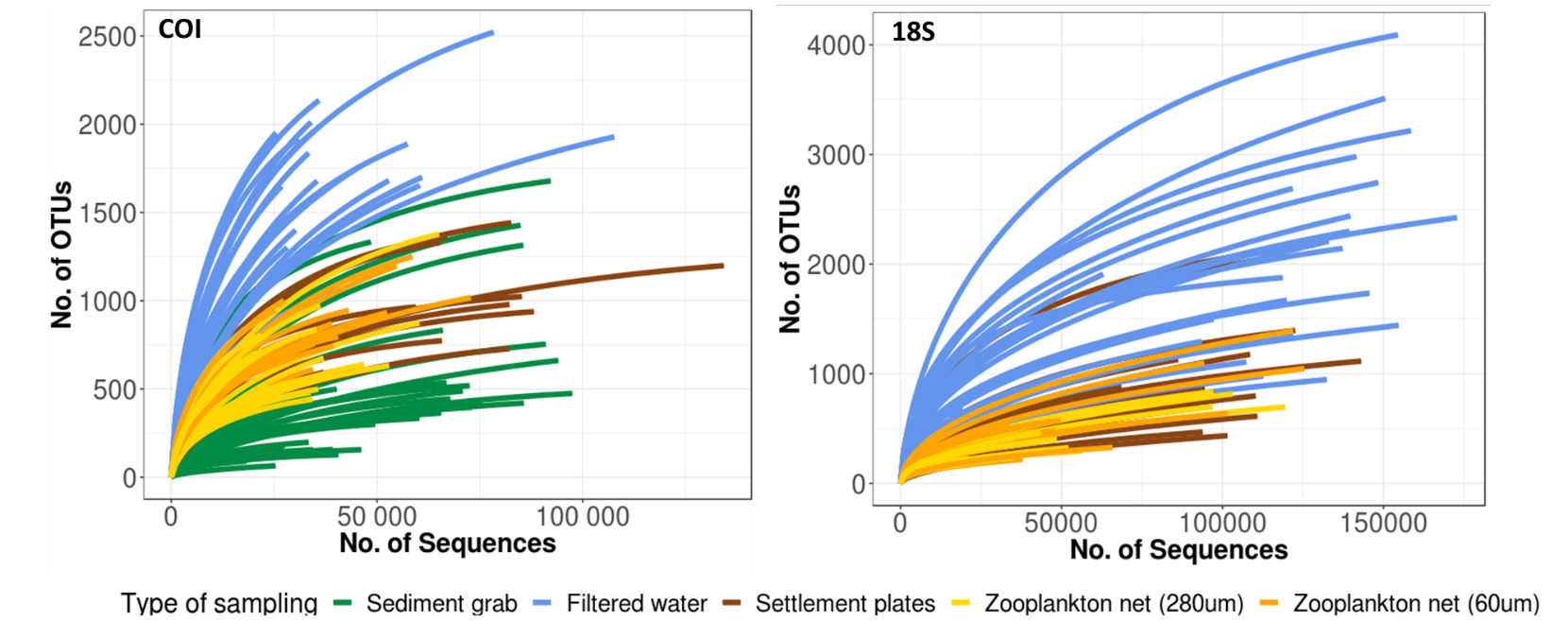

Supplement: Supplementary file 3 [file ECE3-10-2452-s003.tif]

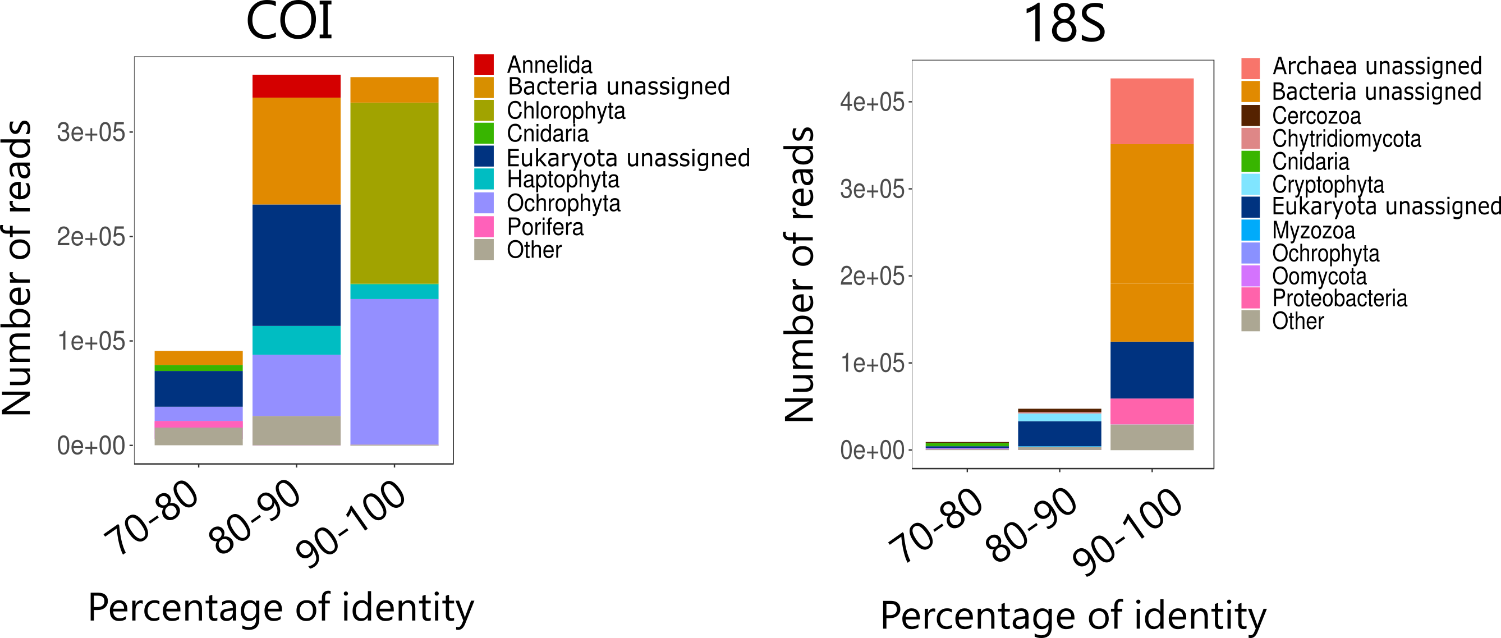

Supplement: Supplementary file 4 [file ECE3-10-2452-s004.tif]

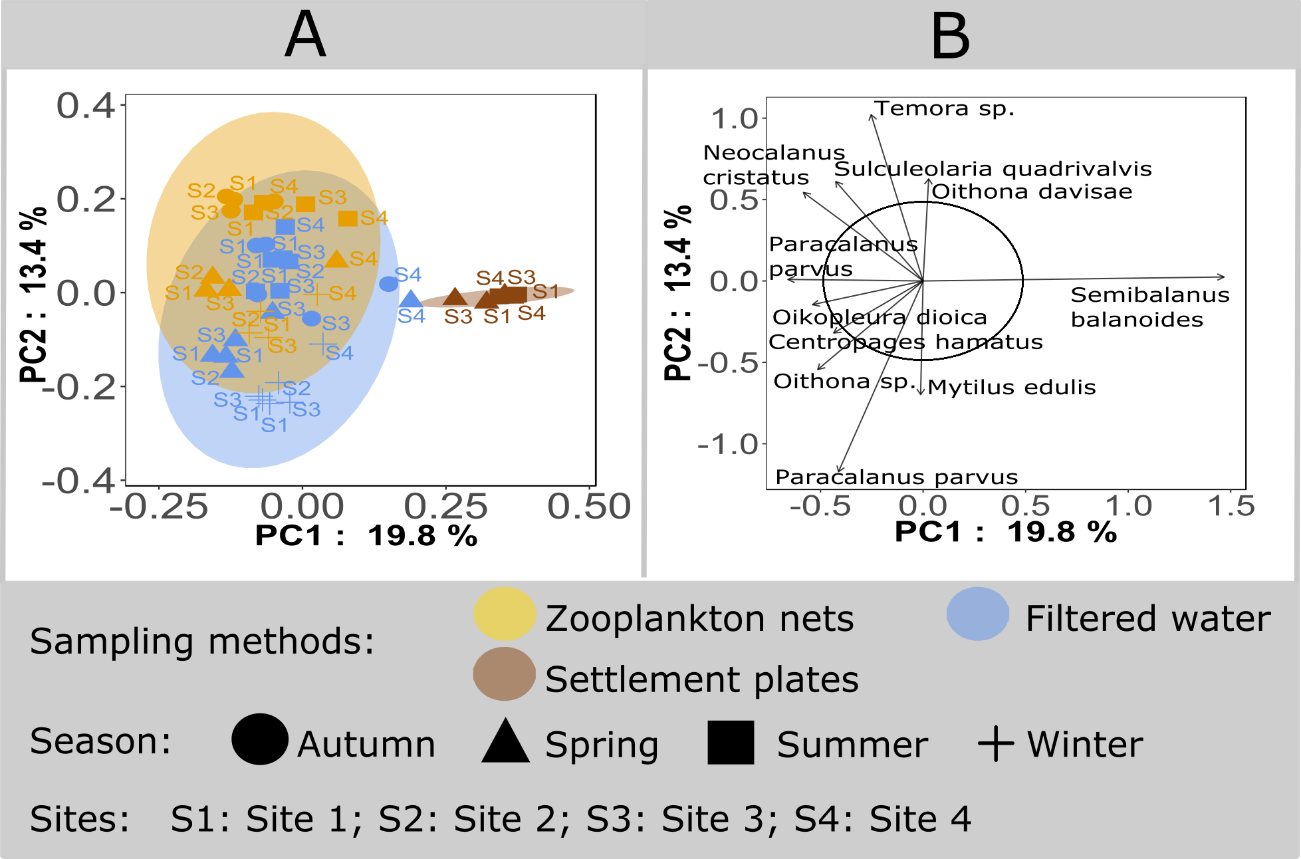

Supplement: Supplementary file 5 [file ECE3-10-2452-s005.tif]

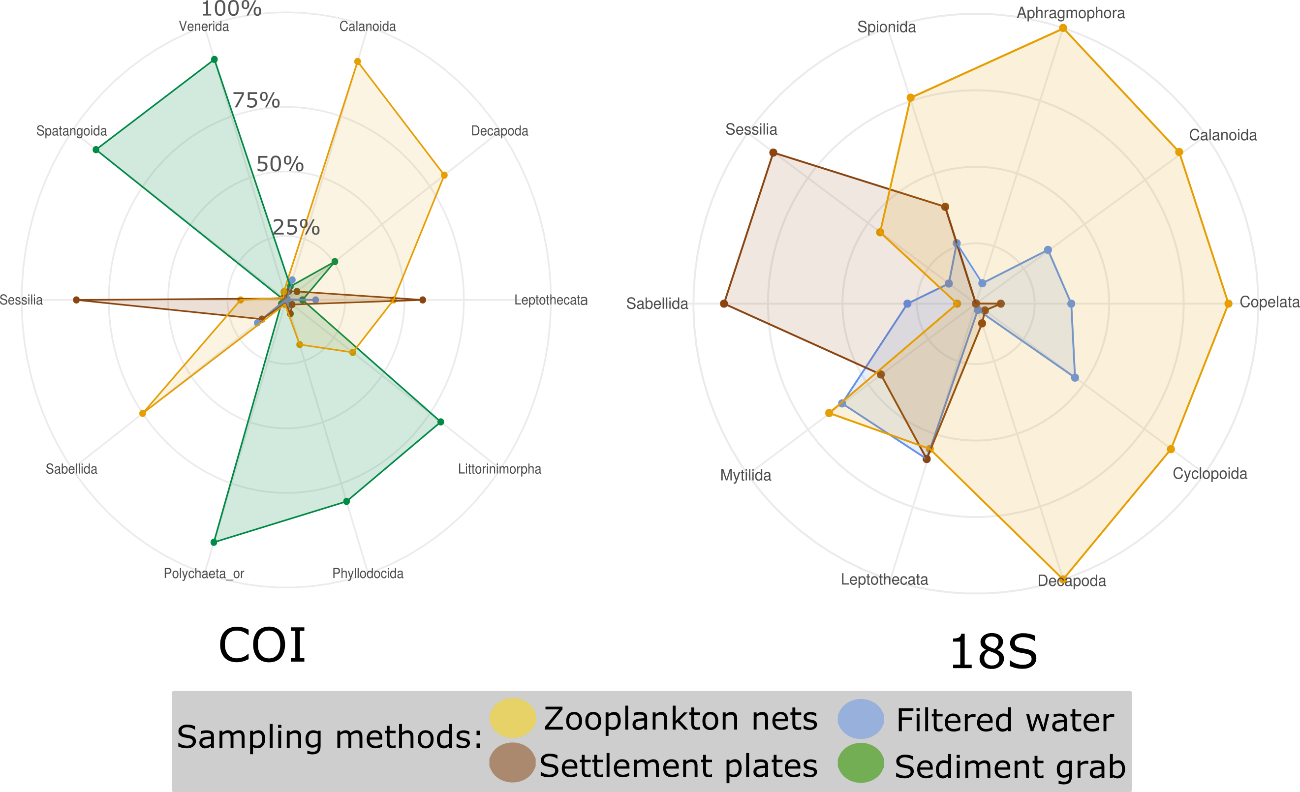

Supplement: Supplementary file 6 [file ECE3-10-2452-s006.tif]

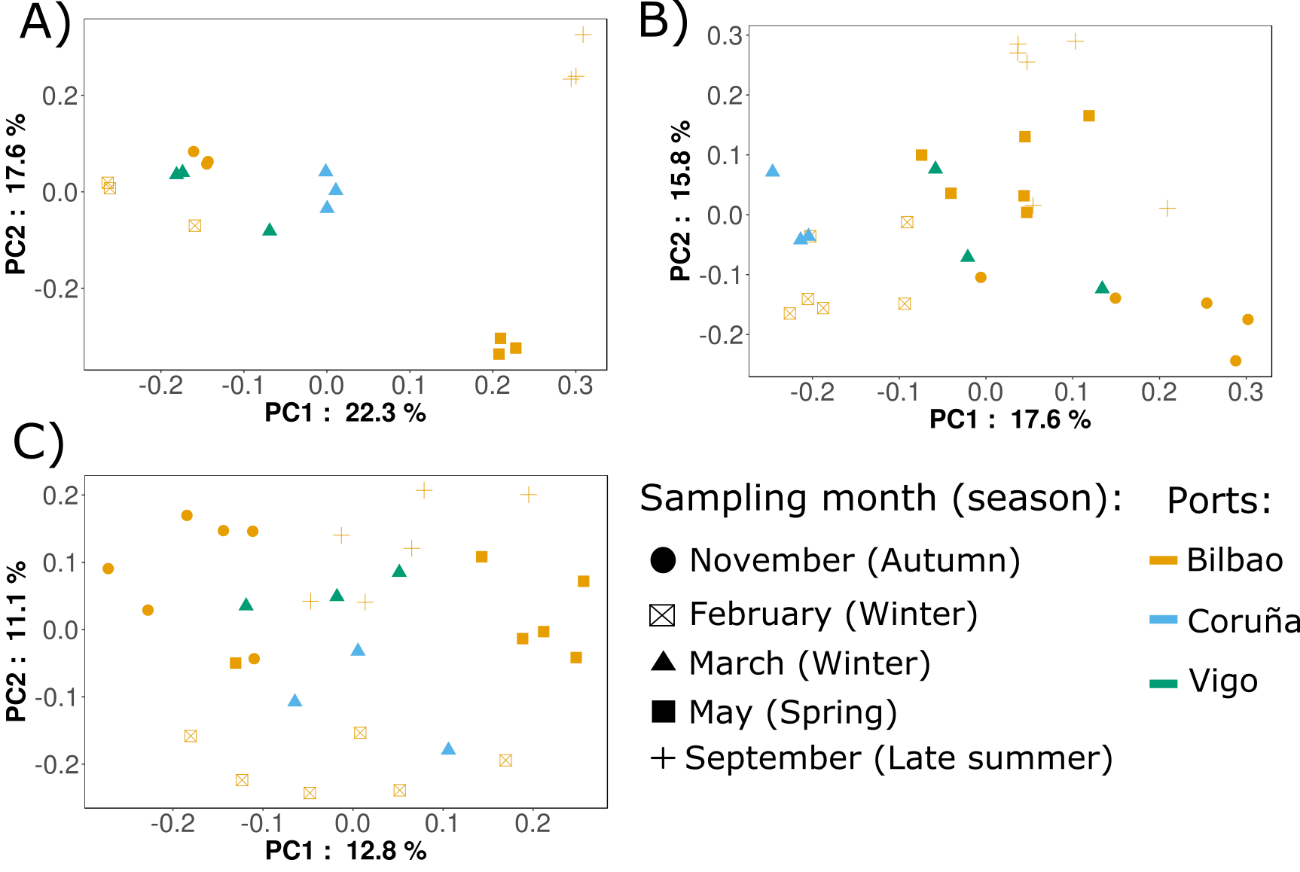

Supplement: Supplementary file 7 [file ECE3-10-2452-s007.tif]
